# Food Processing: Comparison of Different Food Classification Systems

**DOI:** 10.3390/nu14040729

**Published:** 2022-02-09

**Authors:** Taissa Pereira de Araújo, Milena Miranda de Moraes, Cláudia Afonso, Cristina Santos, Sara S. P. Rodrigues

**Affiliations:** 1 Faculty of Nutrition and Food Sciences, University of Porto, 4150-180 Porto, Portugal; milenaferreira@fcna.up.pt (M.M.d.M.); claudiaafonso@fcna.up.pt (C.A.); cristinasantos@fcna.up.pt (C.S.); saraspr@fcna.up.pt (S.S.P.R.); 2 Associated Laboratory ITR, Laboratory for Integrative and Translational Research in Population Health—Institute of Public Health, University of Porto, 4050-600 Porto, Portugal; 3 Associate Laboratory RISE—Health Research Network, Center for Health Technology and Services Research (CINTESIS), University of Porto, 4200-450 Porto, Portugal

**Keywords:** food processing, food classification systems, household surveys, ultra-processed food

## Abstract

The substitution of minimally processed food and culinary home preparations for ready-to-eat products is increasing worldwide, which is overlooked as a cause of concern. The technological developments and the rise in highly processed food availability have introduced the concept of ultra-processed food (UPF). Food classification systems based on processing are now a new basis for epidemiological research. Different results from these classifications might influence conclusions on the population’s consumption of UPF or its association with health outcomes. The aim of this study was to compare classification systems and to find out if their results are comparable when evaluating the extent of high/UPF on the overall diet. Portuguese data from the year 2000 was extracted from the DAFNE-AnemosSoft, and 556 food/beverages items were classified according to five systems. The contribution of UPF was calculated as a percentage of total available amount and discrepancy ranges used for comparisons. Results of UPF availability contributions were: NOVA 10.2%; UNC 15.2%; IFPRI 16.7%; IFIC 17.7%; IARC 47.4%. The highest discrepancy ranges were from alcoholic beverages (97.4%), milk/milk products (94.2%), sugar/sugar products (90.1%), added lipids (74.9%), and cereals/cereal products (71.3%). Inconsistencies among classifications were huge and the contribution from highly/UPF presented high discrepancies. Caution must be taken when comparing and interpreting such data.

## 1. Introduction

The replacement of minimally processed food and culinary home preparations for ready-to-eat products is increasing worldwide, which has been overlooked as a cause of concern [1,2]. These changes in the population’s dietary patterns have been accompanied by increases in the prevalence of obesity and other chronic diseases [3]. Many studies have linked the consumption of highly processed food with several chronic diseases such as cardiovascular disease, cancer, diabetes, metabolic syndrome, anxiety, asthma, autism, in addition to overweight and obesity [4,5,6,7,8,9,10].

Food technologies are commonly developed to preserve and keep food quality attributes [11,12]. Some positive impacts of food processing are, for example, the increase in shelf life and in nutrient bioavailability [13]. However, food processing can also have negative impacts, such as high content of artificial additives and loss of nutrients [14]. Similarly, the content of food in vitamins and minerals, sodium and fibers can be affected by industrial processing [15]. Furthermore, the industry also uses food technology to make food more palatable and with different textures, increasing its consumption [16].

The significance of the techniques and ingredients developed or created by food technology, on the nature of food and on the state of human health, was generally understated previously. Lately, technological developments and the rise in the availability of highly processed food have introduced the concept of ultra-processed food (UPF) to classify these kinds of products [17]. A new approach to industrial food processing and its impact on human health was developed. New food classification systems have been created, due to changes in the scope and purpose of food processing. According to our knowledge, as to the degree of processing, seven different food classification systems have been proposed by researchers worldwide [1,18].

The importance of these food classification systems is that they are a new basis for epidemiological and experimental research and, therefore, for official reports that include dietary guidelines, with the purpose of promoting and protecting health [1,19].

As far as we are concerned, only a few studies performed comparisons between different processing-based food classification systems using the same database [20,21]. One of these studies evaluated the classification of a list of 100 of the most consumed foods [21], the other assessed the contribution of UPF on the overall diet [20], and both used individual-based dietary measures.

Food frequency questionnaires, 24-h recalls, and food diaries are examples of direct methods of individual-based dietary assessments. Individual-level assessment measures food intake, while indirect methods refer to food supply or availability, usually estimated at the national or household level [22]. Household budget survey (HBS) data are particularly relevant once often they are the only tool available to assess food consumption and time trends of populations [23,24]. Since HBS data are freely available and their detailed information allows its use to assess UPF availability contribution, they were used in this study [3].

Due to the variety of food classification systems that consider the degree of food processing, a possible influence of their use in diverse dietary data was hypothesized. The aim of this study was then to compare the food grouping of the different classification systems and to find out if their results were comparable when evaluating the extent of highly/UPF on the overall diet using a database on household food and beverages availability.

## 2. Materials and Methods

### 2.1. Food Classification Systems That Consider the Degree of Processing

Systematic reviews about this subject mentioned seven different food classification systems based on food processing [1,18]. The National Institute of Public Health (NIPH) of Mexico proposed the first classification system in 2007 [25]. In Europe, another one was developed in 2009 by the International Agency for Research on Cancer (IARC) researchers using methodology devised for the European Prospective Investigation into Cancer and Nutrition (EPIC) study [26]. Simultaneously, in Brazil, researchers from the Centre for Epidemiological Studies in Health and Nutrition at the School of Public Health, University of São Paulo focused on the role of industrial processing in nutrition and human health [27]. They created the NOVA classification system that was updated in 2015. This classification defines industrial food processing as methods used by the industry “to make raw foods less perishable, easier to prepare, consume or digest, or more palatable and enjoyable, or else to transform them into food products” [17]. The NOVA classification has been the most used worldwide [9,28,29,30]. In 2011, in Guatemala, a food classification system developed by Asfaw from the International Food Policy Research Institute (IFPRI) was made on previous work examining the contribution of processed food products to food supplies in lower-income countries [31]. In the United States of America (USA) in 2012, another food classification system based on processing level was developed by the International Food Information Council Foundation (IFIC), for determining the contribution of processed foods to nutrient intake in the US diet [32]. In line with the research carried out by Monteiro et al., in the USA in 2015, the University of North Carolina (UNC) developed another classification system, with modified category names and representative foods of the American diet [33]. Finally, in 2018, the Food Standard Australia New Zealand [34] also proposed to dichotomize foods and beverages into not processed or processed. However, this classification does not distinguish processed foods into their degree of processing, impairing measuring highly or UPF.

It was decided not to include systems that do not distinguish highly/ultra-processed food from processed food. Food classification systems with only local/regional applicability were also excluded. Therefore, two classifications were not considered: the FSANZ (Australia and New Zealand, 2018) [34], which classifies food only as unprocessed or processed, not distinguishing ultra or highly processed food; and the NIPH (Mexico, 2007) [25] that is focused on Mexican food products and traditional cuisine. Dietary data were then classified according to the 5 remaining systems: IARC (Europe), NOVA (Brazil), IFPRI (Guatemala), IFIC (USA), and UNC (USA), which main characteristics are summarized in Table 1.

### 2.2. Dietary Data

The present study was performed by analysis of data obtained from the DAFNE-AnemosSoft (http://dafne-anemos.hhf-greece.gr/, accessed on 5 January 2022), where the DAFNE databank—developed in the context of the Data Food Networking (DAFNE) [35] initiative—is accessible. DAFNE databank is a simple and cost-effective tool to monitor food patterns and their socio-demographic determinants across European countries, Portugal included. The available data was compiled through the nationally representative and routinely undertaken Household Budget Surveys (HBS) and thus refers to the overall diet. DAFNE-AnemosSoft data results refer to the mean daily household availability of food and beverages for the overall population of each country. For the present analysis, Portuguese data from the year 2000 was extracted. Such data has previously been used to obtain UPF availability contribution [3], since the information available has enough detail (e.g., yogurts were detailed into natural, liquid, aromatized, with small pieces of cereals or fruits) [35].

The available data is expressed in grams or milliliters per person per day. It was decided to use the absolute quantity instead of converting into total energy once it allows including low or no-calorie foods, especially relevant for ultra-processed food products [36].

### 2.3. Data Analysis

The Portuguese extracted data from 556 food and beverages items were classified according to the five systems under analysis. All items were classified into one of four groups: 1—non-processed or unprocessed or minimally processed food; 2—basic or primary processed food; 3—moderately processed food; 4—highly or ultra-processed food (including the group “prepared food and meals” that was separate in the case of IFIC classification). Such association of items was performed for two independent researchers and a senior one that intervened when inconsistencies arose.

Based on DAFNE-AnemosSoft Portuguese data from year 2000, the total average amount of available food and beverages per day per person was 1620.18 g. For each classification system, the contribution of the different groups, highly/UPF in particular, was calculated as a percentage of this total amount, for the global availability and for each food and beverage category. The 11 categories and 39 subcategories used in data analysis and presentation, followed the DAFNE food classification system (DAFNE Food Classification System, 2005).

The percentage of food availability for each of the 11 categories of food and beverages was compared across the five classification systems using the discrepancy ranges (maximum value—minimum value) and the standard deviations for the average values obtained. For those categories with discrepancy ranges above 50%, the contributions observed for their subcategories were explored to enhance better comparison among classification systems.

## 3. Results

### 3.1. Comparing the Different Grouping among Classification Systems

Table 2 highlights the similarities and differences between the 5 classifications under study. These classification systems differ from each other in the number of groups: IARC and IFPRI classify foods into 3 groups, NOVA and UNC into 4, and IFIC into five groups. In these classifications, group 1 is mainly “raw food”. Group 2, named “processed culinary ingredients” by NOVA, shows similarities to groups “primary processed foods” by IFPRI, and “basic processed food” by UNC. Group 3, named “processed foods” by NOVA, has some similarities to the group “moderately processed foods” by IARC and UNC and “mixtures of combined ingredients” by IFIC. For IFIC, the group “food for preservation”, was also considered in Group 3; although presented as the second group of IFIC classification, the examples refer to processed foods and not culinary ingredients. For NOVA, IARC, and UNC, this group contains foods that are minimally processed, but with the addition of salt, sugar, oils, and other culinary ingredients, in vacuum-package or under a controlled atmosphere. Group 4 is comprised of industrial formulations or foods prepared by the industry, packaged, ready for consumption and with a high content of salt, sugars and fat. For IFIC classification system these foods may contain preservatives used with the aim of promoting safety, flavour, and visual appeal. For these four classification systems, these foods are considered ready-to-eat foods. In the IFIC classification, for better comparison with the other classification systems, both groups (ready-to-eat and prepared meals) were joined in group 4 named highly or ultra-processed food. In this group, the five classification systems have similarities: IARC, IFPRI and UNC have named this group as highly processed foods, and NOVA as ultra-processed food.

**Figure 1:**
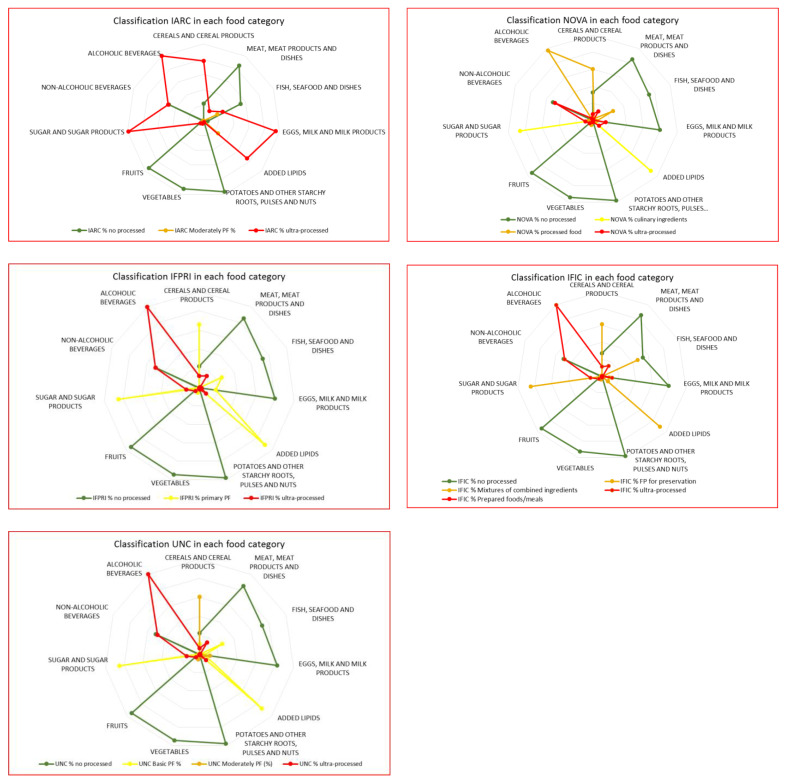
Comparison among food processing classification systems: percentage contribution of their different degree of processing groups by food and beverages categories—Portuguese household budget survey, 2000.

Figure 1 presents the comparison among the percentage contribution obtained for each food and beverages category within the different degree of processing groups proposed by each classification system. In Figure 1 the differences and similarities among classification systems are pictorially highlighted, being clear that IFPRI, IFIC and UNC show a very similar configuration while NOVA and IARC present considerably different ones.

### 3.2. Comparing the Results for Highly/Ultra-Processed Food and Beverages among Classification Systems

For the global diet, the highly/ultra-processed availability contribution according to each one of the classification systems was: NOVA 10.2%, UNC 15.2%, IFPRI 16.7%, IFIC 17.7% and IARC 47.4% (Table 3).

Table 3 shows the precise percentages of highly/UPF in each food and beverages category, by food classification system. The highest discrepancies in results were observed for five categories: Cereals and cereal products, Eggs, Milk, and milk products, Added lipids, Sugar and sugar products, and Alcoholic beverages. The categories with the most homogeneous results among the five classification systems were Meat, Potatoes, Fruits, and Non-alcoholic beverages.

In four of the five most discrepant food categories, it was observed that IARC presented the highest percentages, whereas for alcoholic beverages, only NOVA presented a value (2.6%) different from 100% (Table 3). Details about these five categories are highlighted in Table 4.

For the cereals and cereal products category, the higher resemblance is in the Rice subcategory. Bakery products also showed similar results. The higher discrepancies were observed in subcategories of bread and rolls, pasta and flour, where some systems classify 100% of the products and none of them. (Table 4)

Unanimity is observed in the Eggs subcategory classification, never considered as UPF. The yogurt subcategory is almost fully included as UPF for NOVA, IARC, and IFIC but not for IFPRI and UNC. The IARC system classifies 100% of cheeses and 98.7% of milk as highly processed, while the NOVA classifies only 8.3% of cheeses and 4.2% of milk as UPF. The other classification systems (IFPRI, UNC, IFIC) do not classify any type of cheese as highly/UPF. (Table 4)

For the category of Added lipids, it is visible that all classification systems consider the vegetable fat and margarine subcategory as UPF, except the IFIC, which classifies this subcategory as mixtures of combined ingredients. Relevant discrepancies were observed in the subcategories of lipids of animal origin, seed oils and olive oil. (Table 4)

The highest discrepancy in the category of Sugar and Sugar products was observed in the sugar subcategory, as only the IARC system classifies sugar as highly processed. All other classifications (NOVA, IFPRI, UNC, IFIC) classify this subcategory as basic/primary processed food or culinary ingredients. Sugar products contribution is similar and almost total in all classification systems but not in NOVA, with a much lower contribution (58.4%). (Table 4)

The discrepancy in the category of Alcoholic beverages is concentrated in the NOVA system, which does not classify Wine and Beer as UPF but instead as processed food, while all other classifications consider 100% of these two subcategories as highly/UPF. Unanimity was observed for Spirits, all considered as UPF in the five classification systems. (Table 4)

## 4. Discussion

Analyzing the different classification systems, some similarities were observed in the proposed grouping of foods. However, huge discrepancies were found when analyzing the results of the highly/UPF contribution by using the same dietary database to compare these classification systems. In this study, it was found that the classification system that presented the highest UPF contribution was the IARC (47.4%), followed by the IFIC (17.7%), IFPRI (16.7%) and UNC (15.2%), both with similar values, and NOVA (10.2%) with the lowest contribution value. A recent study, aiming to assess the impact of these classification systems on the association between UPF consumption and cardiometabolic health, compared UPF contribution results by using consumption data from a food frequency questionnaire applied to adult individuals, revealed that the highest consumption share was obtained with the IARC classification (45.9%) and the lowest with NOVA (7.9%) [20]. In agreement with our findings, these results underpin that NOVA, the most used classification, seems to underestimate the contribution of highly/UPF.

Unlike the present findings, a study that evaluated the robustness of food processing classification systems by classifying 100 foods consumed by children, verified that the NOVA system classified most foods as highly/ultra-processed compared to IFIC or UNC. The IFIC system classified less food as highly/ultra-processed, suggesting that this system underestimates the contributions of highly processed foods compared to NOVA and UNC [21]. Nevertheless, this study comprises only a specific age group applies the classification systems to a list of the most consumed foods, not analyzing the contribution for the overall diet, which might explain the disagreement.

Lack of consensus was particularly noticeable while classifying certain food categories and subcategories, namely, cereals and cereal products (for bread and rolls, flour and pasta), milk and milk products (for milk, cheese and yogurts), added lipids (for lipids of animal origin and seed oils), sugar and sugar products (for sugar), and alcoholic beverages (for wine and beer). It is relevant to highlight that the observed discrepancies are very much an all or nothing matter, meaning that the same subcategory of products might be or not be considered as highly/UPF depending on the classification system to be used. Bread is an example that well illustrates such divergences. For IARC bread is always considered highly/UPF because it is a ready-to-eat product (with no need of any domestic preparation). For NOVA and UNC, bread is only a highly/UPF if some specific ingredients (additives, sugar, fats and oils) are added on its production, otherwise, bread is considered as a processed food. For IFPRI and IFIC, bread is never classified as a highly/UPF once they only account for the extent of processing.

A recent paper that provides a qualitative critical overview of the processed-based food classification systems clearly argues for the existence of multiple and different criteria behind the different systems [37]. The identification of the various dimensions used by these systems in the conceptualization of processed foods—extent of change, nature of change, place of processing and purpose of processing—undoubtedly exposes the complexity of such classifications and helps to explain the discrepancies found in the present paper. As Sadler et al. stated, “Food processing and the degree of processing are interpreted in different ways. The classification systems address multiple characteristics of industrial foods as well as eating culture and hence the debates are multifaceted.” [37].

Comparing different dietary databases across diverse food processing classification systems is important to identify discrepancies that exist between them. It is necessary to be aware of such discrepancies when using this type of food classification to ensure the contribution of each food category is not under or overestimated. It is evident that further research is needed and that an effort on a consensual classification system would be of great public health relevance. Results from this study do not allow a clear recommendation on how to choose an appropriate classification system. However, if that was the case, NOVA would likely be chosen—not only for presenting the most conservative approach but also for allowing wider comparisons once it is the most used in the scientific literature.

Despite these discrepancies between classifications, some studies have linked the consumption of highly/ultra-processed foods to lower diet quality, linking processing and nutritional value. Nevertheless, discrepancies between food classification models and nutrient profiles (NP) are common. Labonté et al. [38] found discrepancies between models comparing the degree of strictness and agreement between nutrient profile systems to marketing restrictions of foods and beverages to children in Canada. Labonté et al. [38] considered the extent of food processing (NOVA system) is a novel and interesting avenue, although the categorization of foods according to this method in the original Pan American Health Organization (PAHO) model resulted in much higher discrepancies between team members than the categorization of foods in the other NP models [39]. Mora-Plazas et al. [40] compared two models, the PAHO model and the nutritional profile established by the Chilean food labelling and advertising legislation (Chilean model), and found that most packaged foods in Colombia exceeded critical nutrient thresholds using these NP models.

Apart from being limited by the use of specific data from one single country, there are some strengths in the present study that should be highlighted. First, this analysis used data that is freely available in DAFNE-AnemosSoft, a database that uses nationally representative samples of households. A limitation of this study concerns the use of a database from the year 2000, as since then there may have been changes in processing that may not be captured. Despite this, these data are comparable with other studies, such as that from Monteiro et al. [3], which used this same database and reported the same amount of UPF contribution that was found in the present study. Moreover, for Portugal 2000, the selected database provides detailed information for more than 500 food and beverages items, which allowed for a comprehensive and detailed analysis that has been performed by two independent researchers and a senior one when disagreement occurred. In addition, to the best of our knowledge, this study is the second performing comparison among classification systems on the degree of food processing by analyzing the share of UPF results in the overall diet, and the first that uses indirect consumption data from household availability to perform this evaluation. Furthermore, this study was the first to identify discrepancies among classification systems at the level of food and beverages categories and subcategories.

## 5. Conclusions

Inconsistencies among classifications are huge and thus, results of the contribution from highly/UPF presented high discrepancies. Lack of consensus was particularly noticeable while classifying cereals and cereal products (bread and rolls, flour, and pasta), milk and milk products (milk, cheese, and yogurts), added lipids (lipids of animal origin, and seed oils), sugar and sugar products (sugar), and alcoholic beverages (wine and beer).

The choice of the classification system to be used, clearly influences the conclusions to be taken about the contribution of highly/UPF in the overall diet. Caution must be taken when comparing results from different studies and on its association with health outcomes. An effort on a consensual classification system, based on clear criteria would be of great public health relevance.

## Figures and Tables

**Table 1:** Food processing classification systems: grouping definitions and examples.

Classification Systems	Degree of Processing Groups	Definition	Examples
**IARC—Europe (Slimani et al., 2009)**	**Non-processed foods**	Foods consumed raw without any further processing/preparation, except washing, cutting, peeling, squeezing.	Fruits, non-processed nuts, vegetables, crustaceans, mollusks, fresh juices.
	**Moderately processed foods**	Modest processing and consumed with no further cooking such as dried fruits, raw vacuum-packed or under controlled atmosphere foods. Processed at home and prepared/cooked from raw or moderately processed foods.	Packaged salad, frozen basic foods, extra virgin olive oil, fruits and vegetables canned in water/brine or in own juice, meat and fish cooked from raw/fresh ingredients, or vacuum-packed, deep-frozen.
	**Highly processed foods**	Foods that have been industrially prepared, including those from bakeries and catering outlets, and which require no or minimal domestic preparation apart from heating and cooking.	Bread, breakfast cereals, cheese, commercial sauces, canned foods including jams, commercial cakes, biscuits, and sauces.
**NOVA—Brazil (Monteiro et al., 2016)**	**Unprocessed or Minimally processed food (Group 1)**	Minimal processing is used to preserve the foods, and to make them suitable for storage, facilitate their culinary preparation, enhance their nutritional quality, and easier to digest.	Fresh, chilled, frozen, vacuum-packed vegetables and fruits, grains (cereals), beans and other pulses, roots and tubers, fungi, dried fruits and freshly, unsalted nuts and seeds, spices in general and fresh or dried herbs, corn or wheat flours and fresh or dried pasta, fresh, dried, chilled, frozen meats, poultry, fish, seafood, fresh, pasteurized or powdered milk, yogurt (with no added sugar or other substance), eggs, tea, coffee, drinking water.
	**Processed culinary ingredients (Group 2)**	Highly durable but usually not consumed by themselves.	Salt, sugar and syrups, honey, plant oils, animal fats, corn starch.
	**Processed food (Group 3)**	Ready-to-consume, by themselves or in combinations.	Canned vegetables, cereals or pulses, nuts added with salt or sugar, salted meats, fish preserved in oil or water and salt, canned fruits added sugar, cheeses, and breads.
	**Ultra-processed food (Group 4)**	Formulations of industrial ingredients and substances derived from foods or else created in laboratories, and typically contain little or even no whole foods.	Soft drinks, ‘packet snacks’, ice cream, chocolates, candies, loaf bread, rolls, cookies, cakes, ‘breakfast cereals’ and ‘cereal bars’, ‘energy’ drinks, mayonnaise, frozen products ready for heating (pies, pasta dishes and pre-prepared pizzas), breaded chicken or fish extracts like nuggets, sausage, hamburgers, ‘instant’ soups and noodles...
**IFPRI—Guatemala (Asfaw, 2011)**	**Unprocessed foods**	Not defined	Staple foods such as corn, roots and tubers, vegetables, fruits, meat, fish, beans, eggs, dairy including fresh, dried milk, cream.
	**Primary processed foods**	Not defined	Bread, corn products (including tortillas), vegetable oils, animal fat (including lard and butter) and dairy products like evaporated milk, cheese, yogurt.
	**Highly processed foods**	Foods that have undergone secondary processing into readily edible form, likely to contain high levels of added sugars, fats, or salt.	Pastries, cookies, crackers, sausage and prepared meats, ice cream, frozen desserts, breakfast cereals, confectionery (sweets, chocolate), fat spreads and shortening, pasta products soft drinks, prepared meals like dried soup, formula, and complementary foods.
**IFIC—USA (Eicher Miller et al., 2012)**	**Minimally processed foods**	Foods that retain most of their inherent properties.	Milk, coffee, fruit, vegetables, meat, and eggs, washed and packaged fruits and vegetables and roasted nuts.
	**Foods processed for preservation**	Nutrient enhancement, and freshness are the next level of processing.	Fruit juices, cooked, canned, or frozen vegetables and fruits, canned tuna, and beans.
	**Mixtures of combine ingredients**	Foods containing sweeteners, spices, oils, colours, flavours, and preservatives used to promote safety, taste, and visual appeal.	Breads or rolls, sugars and sweeteners, cheeses, various condiments, and tacos or tortillas, cake mix, jarred tomato sauce, salad dressing, and rice.
	**Ready-to-eat processed**	Foods packaged and mixtures store prepared, containing high amounts of total and added sugars and low amounts of dietary fiber. The highest level of processing.	Soft drinks, sweets, salty snacks, cereal, lunchmeats, breakfast cereal, crackers, ice cream, yogurt, luncheon meats, fruit drinks, and carbonated beverages and alcoholic beverages.
	**Prepared foods/meals**	Foods packaged for ease of preparation such as frozen dinners, entrées and prepared deli foods.	Pizza, prepared meat dishes, and pasta and prepared meals.
**UNC—USA (Poti et al., 2015)**	**Unprocessed foods**	Single-ingredient foods and beverages that have undergone no or very slight modifications that do not change the inherent properties of the food as found in its raw or natural unprocessed form. Specific processes include cleaning, portioning, packaging, removal of inedible fractions, fat reduction, drying, chilling, freezing, or pasteurization. These products are generally single foods that may have components removed but nothing added.	Fresh fruits, vegetables, milk, eggs, unseasoned meat. Poultry without skin and milk without fat.
	**Basic processed foods**	Foods and beverages have been processed but remain as single foods. Processes include extraction, pressing, clarification, refining, purification, and milling. Preservation methods such as canning, milling of grain to remove germ and thus reduce spoilage.	Sugar, oil, or whole-grain flour, concentrating fruit juice to aid storage and transport, fermentation of milk to produce yogurt, or precooking grains (refined-grain flour or pasta), white or instant rice, and fruit or vegetables canned with no additional flavouring steps.
	**Moderately processed foods**	Single minimally or basic processed foods but with the addition of flavour additives (sweeteners, salt, flavours, or fats) for the purpose of enhancing flavour. They are directly recognizable as their original plant or animal sources.	Salted nuts, fruit canned in syrup, or vegetables canned with added salt, whole-grain breads, tortillas, crackers, or breakfast cereals made from whole-grain flour with no added sweeteners or fat.
	**Highly processed foods**	Foods and beverages are multi-ingredient industrially formulated mixtures processed to the extent that they are no longer recognizable as their original plant or animal source.	Ketchup, margarine, mayonnaise, jarred pasta sauce, condiments, dips, sauces, toppings, ingredients in mixed dishes, refined-grain breads, sugar-sweetened beverages (SSBs), cookies, salty snacks, candy, and prepared mixed dishes.

**Table 2:** Comparison among food processing classification systems: summary of differences and similarities.

Classification Systems	Degree of Processing Groups			
	1	2	3	4
**IARC—Europe (2009)**	Non Processed Food		Moderately PF	Highly PF
**NOVA—Brazil (2010, 2016)**	Unprocessed food or Minimally PF	Processed culinary ingredients	Processed food	Ultra-processed food
**IFPRI—Guatemala (2011)**	Unprocessed Food	Primary PF		Highly PF
**IFIC—USA (2012)**	Minimally PF		FP for preser-vation	Prepared foods/meals
			Mixtures of combined ingredients	Ready-to-eat processed
**UNC—USA (2016)**	Unprocessed food	Basic PF	Moderately PF	Highly PF

**Table 3:** Contribution of highly/ultra-processed food and beverages according to different classification systems: global and by food and beverages categories—Portuguese household budget survey, 2000.

	% of Highly/Ultra-Processed Food							
Global and by Food and Beverages Categories	IARC	NOVA	IFPRI	IFIC	UNC	Average	St Dev	Discrepancy Range (DR)
Global	47.40	10.20	16.70	17.70	15.20	21.26	14.97	38.1
CEREALS AND CEREAL PRODUCTS	77.70	7.10	12.10	11.50	6.40	23.48	30.39	**71.3**
MEAT, MEAT PRODUCTS AND DISHES	14.60	12.30	14.60	14.60	14.60	14.14	1.03	2.3
FISH, SEAFOOD AND DISHES	27.00	1.10	1.30	1.20	1.10	6.34	11.55	25.9
EGGS, MILK AND MILK PRODUCTS	94.80	15.30	2.80	12.00	0.60	23.68	40.01	**94.2**
ADDED LIPIDS	74.90	9.80	9.30	0.00	9.30	20.66	30.60	**74.9**
POTATOES, PULSES AND NUTS	2.10	2.10	2.10	2.00	0.70	1.66	0.61	1.4
VEGETABLES	4.00	0.00	0.00	0.00	0.00	0.80	1.79	4.0
FRUITS	5.10	5.10	5.10	4.90	4.90	5.02	0.11	0.2
SUGAR AND SUGAR PRODUCTS	98.80	8.70	13.80	13.80	13.80	29.78	38.65	**90.1**
NON-ALCOHOLIC BEVERAGES	50.20	48.60	50.20	48.70	48.70	49.28	0.84	1.6
ALCOHOLIC BEVERAGES	100.00	2.60	100.00	100.00	100.00	80.52	43.56	**97.4**

**Table 4:** Contribution of highly/ultra-processed food and beverages according to different classification systems by food and beverages subcategories—Portuguese household budget survey, 2000.

	% of Highly/Ultra-Processed Food							
Global and by Food Categories	IARC	NOVA	IFPRI	IFIC	UNC	Average	StDev	Discrepancy Range
CEREALS AND CEREAL PRODUCTS								
Bread and Rolls	100.00	1.20	0.00	0.00	0.00	20.24	44.59	98.80
Bakery products	100.00	100.00	100.00	87.80	100.00	97.56	5.46	12.20
Rice, cereals and products	7.30	7.30	7.30	7.30	7.30	7.30	0.00	0.00
Flour	100.00	0.00	0.00	0.00	0.00	20.00	44.72	100.00
Pasta	100.00	0.00	100.00	100.00	0.00	60.00	54.77	100.00
EGGS, MILK AND MILK PRODUCTS								
Eggs	0.00	0.00	0.00	0.00	0.00	0.00	0.00	0.00
Milk	98.70	4.20	0.00	0.00	0.00	20.58	43.71	94.50
Cheese	100.00	8.30	0.00	0.00	0.00	21.66	43.94	91.70
Yogurts	97.30	96.40	23.60	100.00	5.20	64.50	46.21	94.80
ADDED LIPIDS								
Lipids of animal origin	100.00	10.10	0.00	0.00	0.00	22.02	43.81	89.90
Vegetable fat and margarines	100.00	100.00	100.00	0.00	100.00	80.00	44.72	100.00
Olive oil	30.10	0.00	0.00	0.00	0.00	6.02	13.46	30.10
Seed oils (olive oil excluded)	100.00	0.00	0.00	0.00	0.00	20.00	44.72	100.00
SUGAR AND SUGAR PRODUCTS								
Sugar	100.00	0.00	0.00	0.00	0.00	20.00	44.72	100.00
Sugar products	92.20	58.40	92.20	92.20	92.20	85.44	15.12	33.80
ALCOHOLIC BEVERAGES								
Wine	100.00	0.00	100.00	100.00	100.00	80.00	44.72	100.00
Beer	100.00	0.00	100.00	100.00	100.00	80.00	44.72	100.00
Spirits	100.00	100.00	100.00	100.00	100.00	100.00	0.00	0.00

## Data Availability

All data generated or analyzed during this study are available from the corresponding author on reasonable request.
